# Extrapyramidal signs occurring after sympathetic block for complex regional pain syndrome responding to diphenhydramine: Two case reports

**DOI:** 10.1097/MD.0000000000011301

**Published:** 2018-06-29

**Authors:** Semih Gungor, Rohit Aiyer

**Affiliations:** a Division of Pain Medicine, Department of Anesthesiology, Critical Care and Pain Management, Hospital for Special Surgery—Weill Cornell Medicine, Cornell University; b Department of Psychiatry, Hofstra Northwell Health—Staten Island University Hospital, Staten Island, New York.

**Keywords:** complex regional pain syndrome (CRPS), diphenhydramine, extrapyramidal, sympathetic block, treatment

## Abstract

**Rationale:**

To present an unusual extrapyramidal motor response occurring after a sympathetic block in CRPS and its successful treatment with diphenhydramine.

**Patient concerns:**

Severe pain related to Complex Regional Pain Syndrome type 1 interfering with activities of daily living.

**Diagnoses:**

Complex Regional Pain Syndrome type 1.

**Interventions:**

We report a video case reports of two patients with a diagnosis of CRPS type-I. Both patients exhibited similar presentation of unusual extrapyramidal motor response of the affected limb following lumbar sympathetic block. Both patients were treated with intravenous diphenhydramine to abort the extrapyramidal motor response.

**Outcomes:**

Both patients similarly responded to treatment with intravenous diphenhydramine with abrupt resolution of the motor response.

**Lessons:**

Sympathetic blockade may interfere with the adaptive autonomic reflex circuits of the *motor balance homeostasis* in patients with complex regional pain syndrome. Disinhibition of extrapyramidal system may lead to immediate expression of extrapyramidal signs following the sympathetic block. Diphenhydramine, with its anti-histaminic and anticholinergic properties, may be effective in aborting such extrapyramidal signs, and should be considered as a treatment option in similar cases.

## Abstract 2

Supplemental Digital Content is available in the text

## 1 Introduction

The presence of a variety of motor symptoms and signs related to complex regional pain syndrome (CRPS) has been described in the literature.^[1–3]^ However, based on our search, we have not been able to find any report or literature review on extrapyramidal signs presenting after sympathetic blocks. Also, there has been no report of treatment of such extrapyramidal signs with diphenhydramine (DPH). The authors have obtained written consent to publish this case report from the patients. Institutional IRB approval was obtained for these case reports. These are the case reports of 2 patients with video files showing an unusual presentation of dystonia/movement disorder presenting after lumbar sympathetic blocks, and their successful treatment with IV diphenhydramine.

## 2 Case reports

### 2.1 Case 1

**Figure 1:**
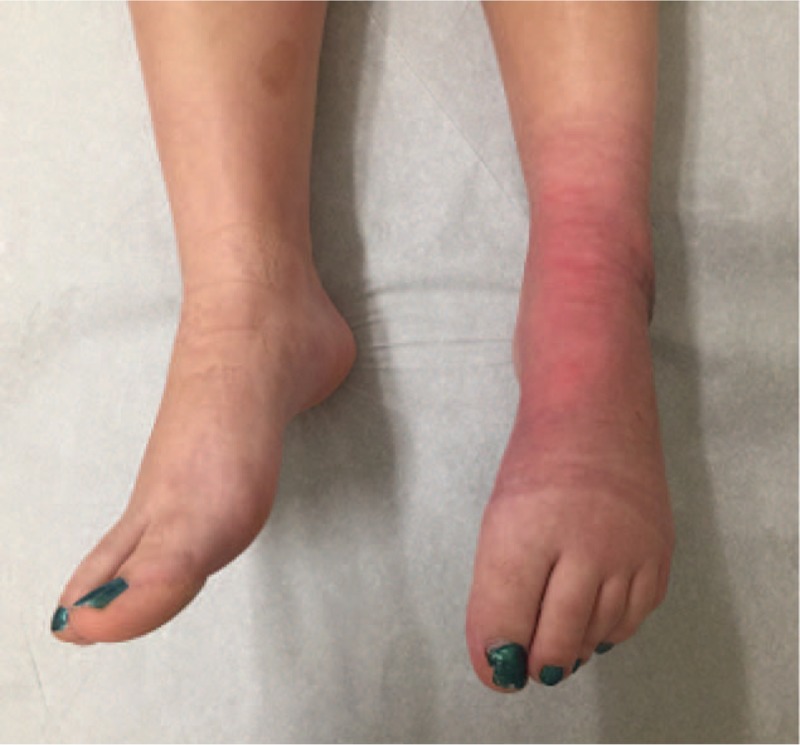
Case 1 with left lower extremity CRPS type-1 and contracture of the foot in plantar-flexed position. CRPS = complex regional pain syndrome.

**Figure 2:**
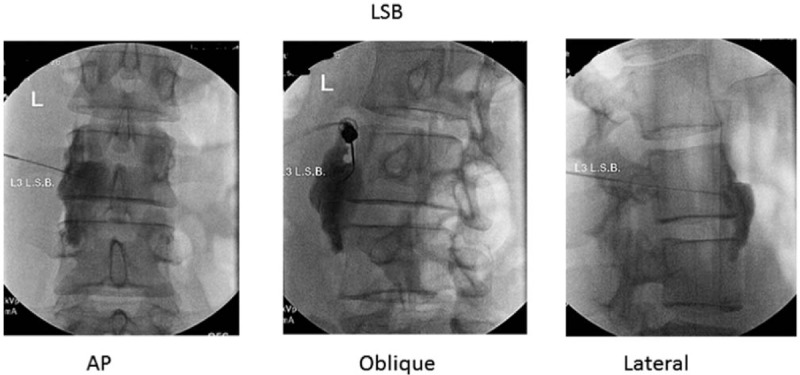
Lumbar sympathetic block technique at L3 level performed for the Case-1.

This is a 22-year-old female patient diagnosed with lower extremity CRPS type-I in the left ankle and foot based on the International Association of the Study of Pain (IASP) Budapest Criteria.^[4]^ The patient did not have any history of psychological disorder, seizure disorder or neurological abnormality. Physical examination in the first presentation also included the contracture of the left foot in plantar-flexed position (Fig. 1), and the only motor presentation of the CRPS was a reproducible tremor of the ipsilateral quadriceps muscles when the left knee was fully extended. The patient did not have any other visible or reproducible involuntary motor movement in her history or physical examination. The patient underwent a diagnostic left lumbar sympathetic block (LSB), followed by 5 additional LSBs with the same technique and medications (Fig. 2). All the procedures were performed under local anesthesia and the patient was given lorazepam 1.5 mg orally as a sedative prior to procedures. In addition, for the 6th block, the patient was given DPH 50 mg IV prophylactically. The patient was placed prone and ipsilateral 30° oblique view was obtained with fluoroscopy. The needle entry point was marked on the skin at the level of L3. After sterile preparation and draping, 3 mL of Lidocaine 1% was used to anesthetize the entry point. 20-gauge 3.5-inch introducer and 25-gauge 6-inch spinal needles were used. The spinal needle was directed to the anterolateral aspect of the L3 vertebral body. There was no paresthesia during advancement of the needles. After negative aspiration for blood and cerebrospinal fluid, 4 mL of Iohexol (180 mgI/mL) contrast was injected under live fluoroscopy to rule out intravascular injection. Appropriate distribution of the contrast in the anterolateral aspect of the L3 vertebral body was verified. Thereafter, 10 mL Bupivacaine 0.5% was injected at 1 mL increments after negative aspiration. Temperature measurements of bilateral plantar skin as well as pulse amplitude of the ipsilateral big toe with pulse oximetry were monitored continuously. Adequate sympathetic blockade was achieved after each block with confirmation of at least 2°C increase from the baseline temperature. Symptomatically, the patient responded well to the series of 6 LSBs with improvement of pain and other CRPS-related signs in the left ankle and foot. However, within 5-minutes of the completion of dose for each first 5 LSBs, the patient developed intense muscle spasms of the entire ipsilateral lower extremity (Video 1). After the first occurrence, various medications were tried to abort these intense spasms including postprocedure IV midazolam, oral cyclobenzaprine and oral baclofen, but they were not effective. The patient eventually responded immediately to IV DPH 50 mg. In the next 4 blocks, the patient was treated similarly with DPH 50 mg IV postprocedure each time with abrupt resolution of muscle spasms. For the sixth LSB, prophylactic treatment of DPH 50 mg IV was given before the procedure and the patient did not show any motor response on the 6th block.

### 2.2 Case 2

**Figure 3:**
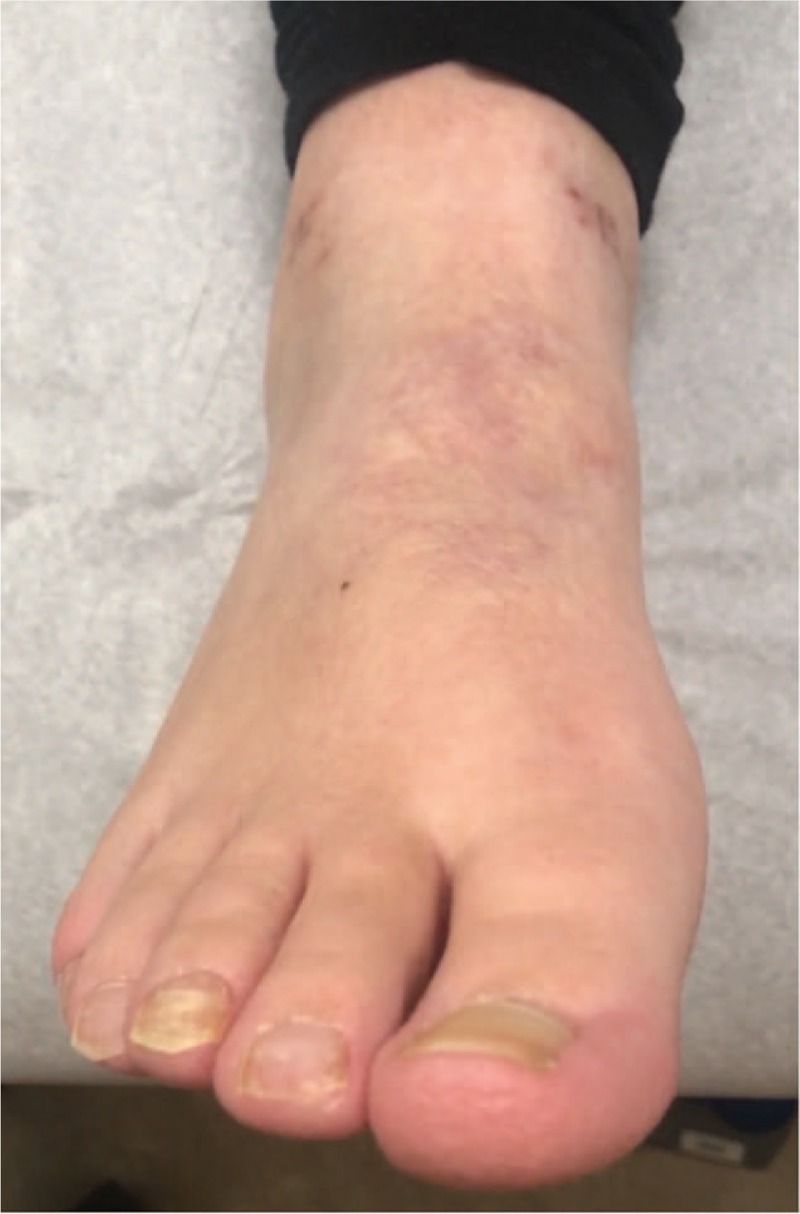
Case 2 with right lower extremity CRPS type-1 with skin color, texture, and trophic changes. The patient also had excessive sweating of the foot. CRPS = complex regional pain syndrome.

This is a 40-year-old female patient diagnosed with lower extremity CRPS type-I in the right ankle and foot, based on the IASP Budapest Criteria.^[4]^ The patient did not have any history of psychological disorder, seizure disorder or neurological abnormality. Physical examination in the first presentation showed the presence of well-healed incision scars from the previous surgeries in the dorsum of the right ankle and foot. There were mild color changes. There were moderate skin texture changes and trophic changes in the nails. The patient had excessive sweating both on inspection and palpation in the entire right foot. There was hyperesthesia and hyperalgesia of the entire dorsum of the foot (Fig. 3). Right foot skin temperature was 3°C colder than the left foot. The patient had 4/5 weakness of the right foot extensors and toe flexors. Right ankle range of motion was moderately limited secondary to pain. The patient did not have any visible or reproducible involuntary motor movement in her history or physical examination. The patient underwent a first LSB with the same technique and medications as described in Case 1, but on the right side. LSB was performed under local anesthesia and the patient was given lorazepam 1.5 mg orally as a sedative prior to procedure. Temperature measurements of bilateral plantar skin as well as pulse amplitude of the right big toe with pulse oximetry were monitored continuously. Adequate sympathetic blockade was achieved after the block with confirmation of at least 2°C increase from the baseline temperature. The patient developed muscle spasms of the entire ipsilateral lower extremity in the recovery room 15 minutes after the completion of the first LSB (Video 2). These muscle spasms were not as intense as the Case-1, but significant enough that the patient was complaining of increased pain and was unable to stand. The patient was initially treated with 2 mg of midazolam IV. The patient did not respond to treatment with midazolam. As the presentation was very similar to Case-1, the patient was treated with IV DPH 50 mg. The patient responded immediately with abrupt resolution of muscle spasms.

## 3 Discussion

CRPS is a pain syndrome associated with combination of sensory, motor, and autonomic changes that usually follow trauma to a limb.^[4,5]^ Patients with this syndrome present with pain and paresthesia, but can also have clinical signs such as edema, changes in temperature and color of skin as well as hyperhidrosis.^[4,5]^ Presentation of motor symptoms and signs of CRPS can include tremors, weakness, fixed dystonic posturing, and myoclonic jerks.^[1]^ CRPS is associated with involuntary motor dysfunction such as dystonia.^[3]^ Verdugo and Ochoa^[3]^ investigated 58 patients with either CRPS I or II with abnormal motor movements. The symptoms and signs included dystonic spasms, coarse postural or action tremor, irregular jerks and choreiform movements.^[3]^ They found that only CRPS type-I cases showed abnormal movements, and thus this could be a potential differentiating feature from type II.^[3]^

There are complex interactions between sensory, motor, and autonomic circuits within the central nervous system (CNS).^[5]^ The autonomic nervous system controls a variety of body functions to maintain homeostasis.^[6]^ Homeostasis is maintained by autonomic reflex circuits that consist of an afferent, a central processing unit in the central nervous system and an efferent component.^[6]^ The afferent autonomic signal predominantly originates from specialized sensors in the periphery and is transmitted to CNS, regulated by various network of autonomic centers in the brain, and a response in return is delivered to 2 branches: the sympathetic and parasympathetic systems.^[6]^ Motor dysfunction seen in patients with CRPS type-I is primarily mediated by central motor neurons in CNS.^[7]^ Maladaptive alterations in the central motor processing circuits of brain with impairment in voluntary motor control at both the affected as well as the unaffected side have been reported in patients with CRPS.^[8]^ Sympathetic nervous system dysfunction is deemed strongly involved in the pathophysiology of CRPS type-I and sympathetic blocks are commonly used in clinical practice for diagnostic and therapeutic purposes despite the scarcity of published evidence for their effectiveness in CRPS.^[9]^ Local anesthetic sympathetic blocks are performed in the sympathetic ganglion chains blocking both pre- and postganglionic sympathetic efferent fibers. Therefore, it is possible that sympathetic blockade may interfere with the adaptive autonomic reflex circuits of the *motor balance homeostasis* in patients with complex regional pain syndrome. Disinhibition of extrapyramidal system may lead to immediate expression of extrapyramidal signs following the sympathetic block observed in our case reports.

Anticholinergic drugs including DPH are commonly used to control extrapyramidal symptoms. DPH has been described in the literature as a clinically effective agent for the treatment of acute dystonia and extrapyramidal symptoms.^[10]^ Truong et al^[11]^ reported 5 patients with idiopathic dystonia who were treated with intravenous DPH 50 mg followed by up to 500 mg/day orally. Their findings showed that 3 patients with jerky clonic dystonia reported symptom relief, while the other 2 patients with tonic dystonia had less relief.^[11]^ Despite its small number, this study nevertheless provides evidence that IV DPH challenge can be a valuable predictor of oral response, and that DPH should be considered as an option to treat clinically patients with idiopathic dystonia, especially those patients presenting lightning jerks.^[11]^ Grañana et al^[10]^ also conducted a similar investigation, they studied 7 patients (6 of whom had generalized dystonia while one patient had craniocervical segmental dystonia). All were treated with both intravenous and oral DPH. While intravenous administration was not a good predictor for favorable oral treatment outcome, all 7 patients did get symptom relief, as all patients elected to continue treatment after completing the protocol.^[10]^

There is still uncertainty of the mechanism of action by which DPH can treat dystonia. While it is known that idiopathic dystonia is associated with alterations in the dopaminergic striatal pathways that are regulated by cholinergic and GABAergic mechanisms, other neurotransmitters may also be involved. An interesting study by Van’t Groenewout et al^[12]^ showed that acute dystonic reactions that are induced in rats through haloperidol injections into the red nucleus were “attenuated by administration of DPH.” Grañana et al^[10]^ further commented on the fact that histamine induced torticollis was antagonized by histamine H1 and H2 receptor blockers, thus illustrating that histamine dysfunction could very possibly be also involved in the pathophysiology of dystonia. Therefore, it is possible that the central anticholinergic and/or antihistaminic effects of DPH are involved in reversing the extrapyramidal signs in cases we presented.

In our Case-1, the differential diagnoses included: psychological reactions, seizure disorder, local anesthetic toxicity, and possible placebo effect. Our assessment was that it was unlikely a psychological reaction as the patient did not have any history of psychological disorder, and she exhibited the reaction every time immediately after the onset of local anesthetic block. She did not respond to pre- and post-LSB administration of antianxiety medications such as benzodiazepines. Moreover, she was unaware of the time when the first dose was given. It is unlikely that this reaction was a manifestation of seizure disorder as the patient did not have history of seizure disorder, and did not respond to treatment with medications that are effective to abort seizure disorder such as benzodiazepines. It is also unlikely that this reaction was related to local anesthetic toxicity as the medication dose given was within normal range. In addition, there was no evidence of intravascular uptake during the LSBs, and the patient did not have generalized reaction other than the affected limb after the first 5 blocks, and did not show other symptoms and signs of local anesthetic toxicity. Placebo response was also unlikely as the patient was unaware that IV DPH had been given before the 6th LSB, after which she did not exhibit the same motor reaction.

In our Case-2, again the differential diagnoses included: psychological reactions, seizure disorder, local anesthetic toxicity and possible placebo effect. Our assessment was that it was unlikely a psychological reaction as the patient did not have any history of psychological disorder. She did not respond to pre- and post-LSB administration of antianxiety medications such as benzodiazepines. It is unlikely that this reaction was a manifestation of seizure disorder as the patient did not have history of seizure disorder, and did not respond to treatment with medications that are effective to abort seizure disorder such as benzodiazepines. It is also unlikely that this reaction was related to local anesthetic toxicity as the local anesthetic dose given was within normal range. In addition, there was no evidence of intravascular uptake during the LSB, and the patient did not have generalized reaction other than the affected limb after the sympathetic block, and did not show other symptoms and signs of local anesthetic toxicity. Placebo response was also unlikely as the patient was unaware of the types of the medications given and she did not respond to IV midazolam, but responded abruptly to IV DPH.

*Limitation*: As opposed to cohort studies or RCTs, case reports have limitations due to the fact that described phenomena may occur by chance; it cannot be replicated in many different subjects. However, these 2 case reports are interesting in that we were able to repeatedly observe the same result on a series of LSBs in one subject, and the second patient with similar presentation also responded to treatment with IV DPH.

## 4 Conclusions

Sympathetic blockade may interfere with the adaptive autonomic reflex circuits of the *motor balance homeostasis* in patients with complex regional pain syndrome. Disinhibition of extrapyramidal system may lead to immediate expression of extrapyramidal signs following the sympathetic block. Diphenhydramine, with its antihistaminic and anticholinergic properties, may be effective in aborting such extrapyramidal signs, and should be considered as a treatment option in similar cases.

## Author contributions

**Conceptualization:** Semih Gungor.

**Data curation:** Semih Gungor, Rohit Aiyer.

**Investigation:** Rohit Aiyer.

**Project administration:** Semih Gungor.

**Resources:** Semih Gungor, Rohit Aiyer.

**Software:** Semih Gungor, Rohit Aiyer.

**Supervision:** Semih Gungor.

**Visualization:** Semih Gungor.

**Writing – original draft:** Semih Gungor, Rohit Aiyer.

**Writing – review & editing:** Semih Gungor, Rohit Aiyer.

## Supplementary Material

SUPPLEMENTARY MATERIAL
